# The impact of the number of stapler firings on anastomotic leakage in minimally invasive rectal surgery: risk factor or technical marker of complexity? A systematic review, meta‑analysis, and metaregression

**DOI:** 10.1007/s10151-026-03364-y

**Published:** 2026-05-26

**Authors:** Stefano Cardelli, Elena Brusa, Giacomo Calini, Tommaso Violante, Alice Gori, Dajana Cuicchi, Luca Stocchi, Matteo Rottoli

**Affiliations:** 1 Surgery of the Alimentary Tract, IRCCS Azienda Ospedaliero-Universitaria Di Bologna, 40139 Bologna, Italy; 2 Department of Medical and Surgical Sciences, Alma Mater Studiorum-University of Bologna, 40139 Bologna, Italy; 3 Division of Colon and Rectal Surgery, Mayo Clinic Florida, Jacksonville, FL USA

**Keywords:** Rectal neoplasms, Minimally invasive surgical procedures, Surgical staplers, Anastomotic leak, Meta-analysis

## Abstract

**Background:**

Anastomotic leakage is a major complication after minimally invasive anterior resection for rectal cancer. The number of stapler firings for rectal transection has been suggested as a modifiable risk factor, but its independent role and optimal threshold remain unclear.

**Objective:**

To evaluate the impact of the number of stapler firings on anastomotic leakage after rectal surgery and to assess the influence of surgical and patient-related variables.

**Data Sources:**

A comprehensive search of PubMed, Cochrane Library, and Ovid MEDLINE was performed.

**Study Selection:**

Studies reporting anastomotic leakage rates stratified by stapler firing count (one, two, or three or more) were included.

**Intervention(s):**

Rectal transection with one or more stapler firings during minimally invasive anterior resection for rectal cancer.

**Main Outcome Measures:**

Incidence of anastomotic leakage according to stapler firing (SF) count.

**Results:**

Twenty two studies including 8,725 patients and 784 anastomotic leakage events were analyzed. A single stapler firing was associated with a significantly reduced risk of anastomotic leakage compared with two stapler firings (odds ratio 0.57, 95% confidence interval 0.39–0.83), three or more stapler firings (odds ratio 0.28, 95% confidence interval 0.16–0.51), and two or more stapler firings (odds ratio 0.46, 95% confidence interval 0.34–0.63). The risk of anastomotic leakage was lower with fewer than three stapler firings compared with three or more stapler firings (odds ratio 0.38, 95% confidence interval 0.30–0.48). Meta-regression identified low rectal transection and preoperative radiotherapy as significant effect modifiers.

**Limitations:**

All included studies were observational, introducing potential bias and increased heterogeneity and the certainty of evidence was low across all comparisons.

**Conclusions:**

Three or more stapler firings were consistently associated with a higher risk of anastomotic leakage. However, because the certainty of evidence was low and owing to exploratory meta-regression findings, stapler firing number appears to reflect operative complexity more than a definitively established independent causal risk factor.

**Supplementary Information:**

The online version contains supplementary material available at 10.1007/s10151-026-03364-y.

## Introduction

Anastomotic leakage (AL) is a major complication following anterior resection for rectal cancer, with an incidence ranging from 2% to 15% [1–3]. The increased morbidity, possible need for reoperation, and extended length of hospital stay result in an additional health care burden and increased direct hospital costs by 50% to 300% per patient [4, 5].

Several patient- and procedure-related risk factors for AL have been identified. Among these, the technical aspects of rectal transection have gained increasing attention. In particular, the number of stapler firings (SFs) required for rectal transection has been independently associated with an elevated risk of leakage [1, 6]. Consequently, ASCRS guidelines and international expert consensus statements recommend minimizing the number of SFs, whereas the Chinese consensus explicitly advises limiting the transection to one or two firings [7–9].

However, the number of SFs is often correlated with other established risk factors for AL, such as male sex, narrow pelvis, and low tumor location, introducing potential collinearity [10–13]. Although several retrospective studies have explored the impact of SFs using multivariable models, this relationship tends to be lost when data are aggregated in meta-analyses. Furthermore, there is currently no consensus on a specific threshold number of SFs that increases the risk of AL, which limits the ability to provide clear intraoperative guidance for safe anastomotic construction.

This study aims to evaluate differences in AL rates across multiple SFs cohorts during minimally invasive rectal resections and to assess how the number of SFs correlates with other recognized risk factors for AL. We hypothesize that an increased number of SFs during rectal transection correlates with a higher risk of AL. Understanding this relationship is clinically important for deciding whether efforts should be directed toward actively reducing SFs with single-firing techniques or whether the number of firings should mainly be seen as a marker of operative complexity rather than an independent risk factor.

## Materials and methods

This meta-analysis was registered in PROSPERO (ID: CRD420251062761). The study was conducted and reported in accordance with the PRISMA 2020 guidelines [14] for systematic reviews and meta-analyses.

### Eligibility criteria

We included randomized controlled trials, prospective or retrospective cohort studies, and case–control studies examining patients undergoing rectal resection (laparoscopic or robotic) with a primary anastomosis through a double-stapled technique. Studies were eligible if they reported the number of SFs used for rectal transection—classified as 1, 2, or ≥ 3 firings—and reported the incidence of AL. AL definitions, grades, and management were evaluated for each individual study. Pure open surgery cohorts and conversions were excluded, whereas mixed minimally invasive surgery (MIS)/open cohorts were retained if they met the inclusion criteria. Pediatric populations or the absence of AL stratification by the number of SFs used were also excluded.

### Data sources and search strategy

A comprehensive search of PubMed, Cochrane Library, and Ovid MEDLINE was conducted using the following terms to identify articles for meta-analysis: “laparoscopic,” “laparoscopy,” “robotic,” “robot,” “rectal resection,” “low anterior resection,” “proctectomy,” “anastomotic leakage,” “anastomotic leak,” “stapler number,” and “stapler firings.” Only English-language full-text articles were considered. No grey literature or trial registries were searched. No time restriction was applied. The literature search was last updated in May 2025, according to the study timeline.

### Study selection and data extraction

Two authors (S.C. and E.B.) independently searched the three bibliographic databases. Each author then selected a subset of relevant studies on the basis of the PICO (Population, Intervention, Comparator, and Outcome) eligibility criteria.

**Fig. 1:**
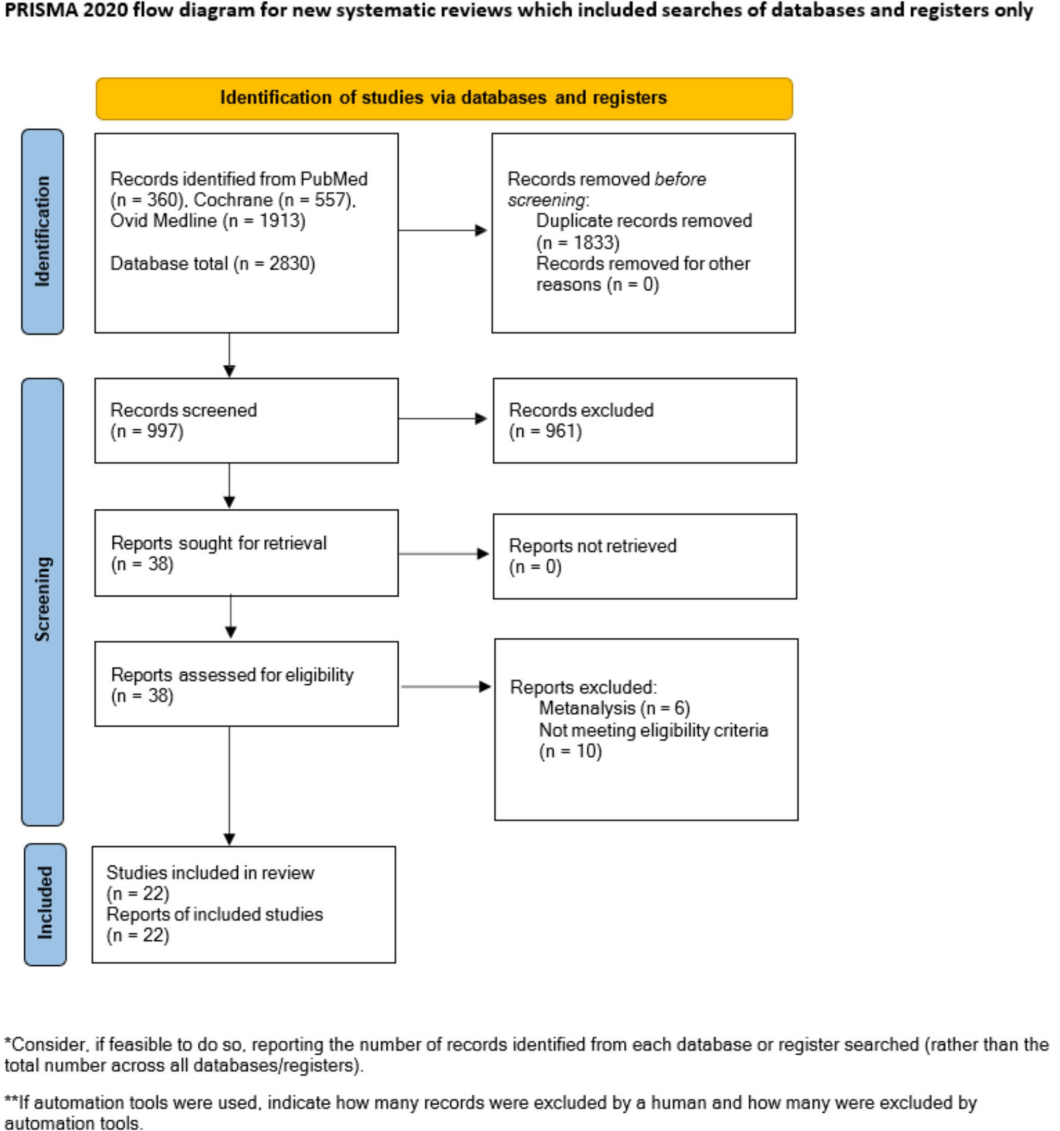
PRISMA 2020 flow diagram of the study search and screening process

The study population included patients over 18 years old who underwent rectal resection with primary anastomosis. The intervention involved constructing a primary anastomosis using a double-stapled technique that required linear stapled rectal transection with one or more SFs. The number of SFs used was compared, and the primary outcome was the AL rate per SF group. Studies meeting the selection criteria were then assessed through title, abstract, and full-text review. After individually selecting the studies, the two authors compared their results to finalize the selection of publications to be included in the present analysis. Disagreements were resolved through discussion and consultation with a third author (G.C.). Figure 1 reports the PRISMA 2020 flow diagram of the study search and screening process.

Key details from each study were documented in a custom-designed meta-analysis form using Excel 2016. Variables recorded included: authors, year of publication, design, total number of patients, surgical approach, and number of SF for each category, including total and AL events. When available, we also extracted descriptive information on linear stapler cartridge length (e.g., 45 mm versus 60 mm), stapler load type (e.g., blue, green, purple/black), air-leak test rate, diverting loop ileostomy rate, and AL definitions and classifications.

We conducted a qualitative assessment of the studies using the validated Methodological Index for Non-Randomized Studies (MINORS) [15].

The primary endpoint of this study was to compare the AL rates among five predefined categories on the basis of the number of SFs for rectal transection: 1 versus 2, 1 versus ≥ 3, 2 versus ≥ 3, 1 versus ≥ 2, and < 3 versus ≥ 3 SF. The secondary endpoint was the influence of established risk factors on the correlation between the number of SFs and the AL rate.

### Risk of bias assessment

All studies were independently evaluated by two authors (S.C. and E.B.) using the ROBINS I tool [16]. A third author (G.C.) confirmed the final determination after discussion. The Grading of Recommendations Assessment, Development, and Evaluation (GRADE) assessment was used to investigate the quality of the evidence for each outcome [17]. The certainty of the evidence was deemed high, moderate, low, or very low.

### Statistical analysis

Meta-analyses were primarily conducted using random-effects models (inverse-variance method with REML estimator for τ^2^), given the expected clinical and methodological heterogeneity across included observational studies. Fixed-effect Mantel–Haenszel models were additionally performed as supportive sensitivity analyses. Results were expressed as odds ratio (OR) with 95% confidence interval (CI) and statistical significance was defined as two-sided α = 0.05. Between-study heterogeneity was assessed using the I^2^ statistic (I^2^ ≥ 75% was considered high, 50–74% substantial, 25–49% moderate, and I^2^ < 25% low heterogeneity) and publication bias was evaluated using the trim-and-fill method (L-estimator) along with visual inspection of funnel plot symmetry. Egger’s regression test was additionally performed only for comparisons including at least 10 studies, whereas for comparisons with fewer than 10 studies publication bias was explored descriptively without formal asymmetry testing.

Mixed-effects meta-regression models were used to explore potential sources of heterogeneity, with residual heterogeneity and explained variance (R^2^) reported. Meta-regression analyses were performed using study-level covariates selected a priori on the basis of biological plausibility, established association with anastomotic leakage, and availability across included studies; these included low rectal transection height, preoperative radiotherapy, body mass index, and male sex. To address heterogeneity in anastomotic leak definitions, we repeated the meta-analysis, restricting inclusion to studies that explicitly adopted the International Study Group of Rectal Cancer (ISREC) definition of anastomotic leakage. All statistical analyses were performed using R (version 4.3.2).

## Results

### Study selection and characteristics

A total of 2830 records were identified through database searches (PubMed, Cochrane, Ovid MEDLINE), with 1830 duplicates removed. After screening 997 unique records, 22 studies [10–13, 18–35] were ultimately included in the meta-analysis involving 8725 patients and 784 AL events. The definitions, grading, and treatment of AL are presented in Table 1. The study selection process is shown in the PRISMA 2020 flow diagram (Fig. 1).

**Table 1:** Definition and grade of anastomotic leak

Study	Year	Definition of leak	Grade of leak	Management of leak
Ito [33]	2008	Discharge of gas, pus, or feces from the drain or wound; discharge of pus per rectum; or rectovaginal fistula	NA	NA
Kim [32]	2009	Discharge of gas, pus, or feces through the pelvic drain, peritonitis, or pus discharge through the rectum	100% C (*n* = 17)	Diverting ileostomy (*n* = 13), end colostomy (*n* = 2), segmental colonic resection with reanastomosis (*n* = 1), irrigation and drainage (*n* = 1)
Choi [31]	2010	Pus or fecal discharge from the drain; increased temperature (> 38 °C) or leukocyte count, or peritoneal irritation sign on physical examination; or rectovaginal fistula or abscess in the pelvic cavity	6% B (*n* = 1), 94% C (*n* = 15)	Diverting ileostomy (*n* = 13), transanal irrigation and primary repair (*n* = 3)—two of the three cases subsequently had an additional ileostomy
Huh [30]	2010	Pus or fecal discharge through the pelvic drain, demonstrating abscesses and/or fluid or air bubbles surrounding the anastomosis	11% B (*n* = 2), 89% C (*n* = 17)	Loop-ileostomy (*n* = 16), loop-transverse colostomy (*n* = 1), conservative treatment (*n* = 2)
Akiyoshi [13]	2011	Gas or fecal discharge from the pelvic drain or vagina, fecal peritonitis, extravasation of endoluminally administered water-soluble contrast on radiography, abscess at the level of the anastomosis, and fluid/air bubbles surrounding the anastomosis on computed tomography (CT)	62% B (*n* = 8), 38% C (*n* = 5)	Loop-ileostomy (*n* = 4), percutaneous drainage (*n* = 4), drainage per anus (*n* = 3), transverse colostomy (*n* = 1) antibiotics (*n* = 1)
Yamamoto [29]	2012	Pelvic abscess, fecal or purulent discharge from a drainage tube or wound, peritonitis	50% B (*n* = 3), 50% C (*n* = 3)	Diverting ileostomy (*n* = 3), conservative treatment (*n* = 3)
Kang [35]	2013	Abdominal pain, fever, and pus or fecal discharge from the pelvic drainage tube	100% C (*n* = 5)	Hartmann’s procedure (*n* = 2), re-anastomosis with a diverting loop ileostomy (*n* = 3)
Kawada [28]	2014	Abdominal pain, fever, pus, or fecal discharge from the pelvic drain, peritonitis, and pelvic abscess	63% B (*n* = 12), 37% C (*n* = 7)	Diverting ileostomy (*n* = 6), colostomy (*n* = 1), percutaneous drainage (*n* = 1), transanal drainage (*n* = 7), antibiotics (*n* = 4)
Braunschmid [12]	2017	International Study Group of Rectal Cancer (ISREC)	11% B (*n* = 2), 89% C (*n* = 16)	Ileostomy (*n* = 8), Hartmann’s procedure (*n* = 4), repair of the anastomosis (*n* = 2), redo anastomosis (*n* = 1), transanal endoscopic vacuum therapy (*n* = 3)—one of the three cases underwent additional laparotomy and lavage for persistent peritonitis
Lee [11]	2017	Discharge of pus or feces from the pelvic drain and signs of peritonitis including abdominal pain, tenderness, fever, or leukocytosis	100% B (*n* = 1)	Antibiotics (*n* = 1)
Tanaka [27]	2017	Abdominal pain, a fever and the discharge of feces, gas or pus from the pelvic drain	6% A (*n* = 2), 42% B (*n* = 14), and 52% C (*n* = 17)	NA
Tejedor [26]	2019	NA	NA	NA
Zhou [25]	2019	International Study Group of Rectal Cancer (ISREC)	70% B (*n* = 21), 30% C (*n* = 9)	NA
Sakamoto [24]	2020	Abdominal pain, fever, and pus or fecal discharge from the pelvic drainage tube	45% B (*n* = 5), 55% C (*n* = 6)	Diverting ileostomy (*n* = 6), conservative treatment (*n* = 5)
Degiuli [23]	2021	International Study Group of Rectal Cancer (ISREC)	18.5% A (*n* = 91), 27.5% B (*n* = 136), 54% C (*n* = 267), and 10.5% grade missing (*n* = 58)	Ileostomy, colostomy, endoscopic stent or clip placement, endoscopic vacuum therapy, or percutaneous pelvic drain positioning
Brisinda [22]	2022	International Study Group of Rectal Cancer (ISREC)	43% B (*n* = 26), 57% C (*n* = 35)	Stoma (*n* = 35), radiological drainage (*n* = 6), antibiotics (*n* = 4), or endoluminal vacuum therapy (*n* = 16)
Eriksen [21]	2022	International Study Group of Rectal Cancer (ISREC)	NA	NA
Kishiki [20]	2022	Local or generalized peritonitis and discharge of pus or feces from the drain	NA	NA
Cai [19]	2023	NA	NA	NA
Vignali [18]	2023	International Study Group of Rectal Cancer (ISREC)	10% A (*n* = 2), 14% B (*n* = 3), 76% C (*n* = 16)	Ileostomy with anastomotic repair (*n* = 6), Hartmann’s procedure (*n* = 5), or peritoneal lavage and drain (*n* = 5)
Fu [34]	2023	NA	NA	NA
Cardelli [10]	2025	International Study Group of Rectal Cancer (ISREC)	70% B (*n* = 39), 30% C (*n* = 17)	Percutaneous drainage of a peri-anastomotic abscess (*n* = 33), emergent reoperation (*n* = 17), or antibiotics (*n* = 6)

NA indicates that the original study did not report the definition, grading, or management of anastomotic leakage

Among the studies examining minimally invasive rectal resections, five reported a mixed population that included an open surgical approach [12, 22–25]. All the included studies indicated the use of the double-stapling technique for anastomotic construction and reported the number of SFs at different cut-off points. Patient-level and study-level characteristics, such as surgical approach, MINORS quality scores, and subgroup stratifications, are presented in the main characteristics table (Table 2).

**Table 2:** Summary of main characteristics of studies included in meta-analysis

Study	Year	Design	Surgical approach	No. of patients	No. AL	MINORS	1 SF		2 SFs		< 3 SF		≥ 2 SF		≥ 3SF	
							tot	AL	tot	AL	tot	AL	tot	AL	tot	AL
Ito [33]	2008	Retrospective	Laparoscopic	180	9	16	NA	NA	NA	NA	153	5	NA	NA	27	4
Kim [32]	2009	Prospective	Laparoscopic	270	17	19	92	1	146	13	238	14	178	16	32	3
Choi [31]	2010	Prospective	Laparoscopic	145	14	19	NA	NA	NA	NA	103	7	NA	NA	42	7
Huh [30]	2010	Prospective	Laparoscopic	206	17	17	NA	NA	NA	NA	162	11	NA	NA	44	6
Akiyoshi [13]	2011	Prospective	Laparoscopic	363	13	18	105	1	227	12	332	13	258	12	31	0
Yamamoto [29]	2012	Prospective	Laparoscopic	111	6	19	55	2	40	3	95	5	56	4	16	1
Kang [35]	2013	Retrospective	Laparoscopic	189	5	19	157	4	NA	NA	NA	NA	32	1	NA	NA
Kawada [28]	2014	Retrospective	Laparoscopic	154	19	16	NA	NA	NA	NA	131	13	NA	NA	23	6
Braunschmid [12]	2017	Retrospective	Laparoscopic/Open	382	18	15	223	6	128	6	351	7	159	12	31	6
Lee [11]	2017	Retrospective	Laparoscopic	128	1	17	40	0	82	1	122	1	88	1	6	0
Tanaka [27]	2017	Prospective	Laparoscopic	390	33	16	199	13	163	15	362	28	196	20	28	5
Tejedor [26]	2019	Retrospective	Robotic	196	10	14	NA	NA	NA	NA	133	4	NA	NA	63	6
Zhou [25]	2019	Retrospective	Laparoscopic/open	288	30	16	NA	NA	NA	NA	177	12	NA	NA	111	18
Sakamoto [24]	2020	Retrospective	Laparoscopic/open	157	11	15	NA	NA	NA	NA	148	8	NA	NA	9	3
Degiuli [23]	2021	Retrospective	Laparoscopic/robotic/open	3057	298	17	1794	143	NA	NA	NA	NA	1263	155	NA	NA
Brisinda [22]	2022	Retrospective	Laparoscopic/open	583	61	13	351	19	NA	NA	NA	NA	232	42	NA	NA
Eriksen [21]	2022	Prospective	Laparoscopic/robotic	344	44	18	35	2	263	30	298	32	309	42	46	12
Kishiki [20]	2022	Retrospective	Laparoscopic	201	16	12	NA	NA	NA	NA	179	11	NA	NA	22	5
Cai [19]	2023	Retrospective	Laparoscopic	260	37	14	NA	NA	NA	NA	212	25	NA	NA	48	12
Vignali [18]	2023	Prospective	Laparoscopic	290	21	16	164	8	104	10	268	18	126	13	22	3
Fu [34]	2023	Retrospective	Laparoscopic	328	48	16	NA	NA	NA	NA	270	34	NA	NA	58	14
Cardelli [10]	2025	Retrospective	Robotic	503	56	17	164	15	245	28	409	43	339	41	94	13

Risk of bias was evaluated using the ROBINS-I tool (Supp. Fig. S1). Among all included studies, which mostly showed a moderate risk, three had a serious risk of bias owing to confounding and selection of the reported result [26, 27, 32].

### Anastomotic Leakage by Number of Stapler Firings

#### 1 versus 2

**Fig. 2:**
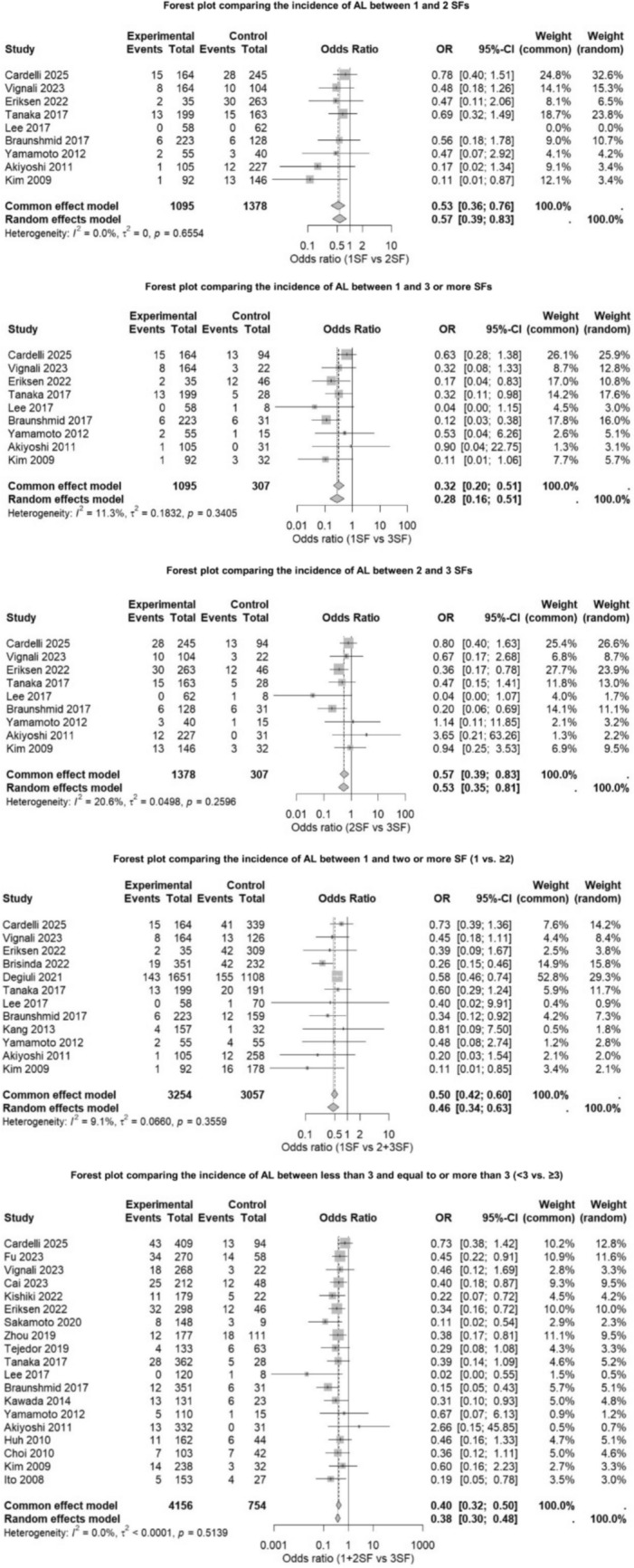
Forest plots comparing the incidence of AL between SFs

Nine studies [10–13, 18, 21, 27, 29, 32], involving 2781 patients and 166 AL events, were analyzed to compare outcomes between one and two SFs. The fixed-effect model revealed a significantly lower risk of AL with a single firing (OR: 0.53, 95% CI: 0.36–0.76, *p* = 0.0006), a finding supported by the random-effects model (OR: 0.57, 95% CI: 0.39–0.83, *p* = 0.0034). Heterogeneity was minimal (I^2^ = 0%) (Fig. 2). Although Egger’s test was not conducted owing to the limited number of studies, potential publication bias was assessed using the trim-and-fill method. Four additional studies were imputed, and the adjusted OR remained significant (OR: 0.64, 95% CI: 0.45–0.92, *p* = 0.0147), confirming the robustness of the association (Supp. Fig. S2).

#### 1 *versus ≥ 3*

Overall, nine studies [10–13, 18, 21, 27, 29, 32], involving 2781 patients and 91 cases of AL, were included in the meta-analysis comparing one versus three SFs. Both fixed- and random-effects models showed a significantly lower risk of AL with a single SF. The fixed-effect model produced an OR of 0.32 (95% CI: 0.20–0.51, *p* < 0.0001), while the random-effects model showed an OR of 0.28 (95% CI: 0.16–0.51, *p* = 0.0001). Between-study heterogeneity was low (I^2^ = 9.0%) (Fig. 2). Funnel plot asymmetry was examined using the trim-and-fill method, which indicated the potential presence of one missing study. After adjustment, the combined effect remained statistically significant (Supp. Fig. S3).

#### 2 *versus ≥ 3*

In total, nine studies [10–13, 18, 21, 27, 29, 32], including 2781 patients and 161 AL events, were examined to compare outcomes between two and three SFs. The results showed a significantly lower AL risk with two firings. The fixed-effect model estimated an OR of 0.57 (95% CI: 0.39–0.83, *p* = 0.0081), while the random-effects model reported an OR of 0.53 (95% CI: 0.35–0.81, *p* = 0.0067). Heterogeneity was minimal (I^2^ = 0%) (Fig. 2). Funnel plot asymmetry was assessed using the trim-and-fill method, which indicated the possible absence of one study. After adjustment, the association remained significant (OR: 0.54, 95% CI: 0.36–0.82, *p* = 0.0036), confirming the stability of the findings (Supp. Fig. S4).

#### 1 *versus ≥ 2*

In total, 12 studies [10–13, 18, 21–23, 27, 29, 32, 35], involving 6610 patients and 573 AL were analyzed to compare single versus multiple (≥ 2) SFs during rectal transection. The meta-analysis showed a significantly lower risk of AL in patients who underwent transection with a single SF. The fixed-effect model estimated an OR of 0.50 (95% CI: 0.42–0.60, *p* < 0.0001), while the random-effects model produced an OR of 0.46 (95% CI: 0.34–0.63, *p* < 0.0001). Heterogeneity was low to moderate (I^2^ = 23.9%) (Fig. 2). No significant funnel plot asymmetry was observed (Egger’s test: *p* = 0.1034). The trim-and-fill method indicated potential unpublished studies; however, the adjusted effect size remained significant (OR: 0.59, 95% CI: 0.42–0.85, *p* = 0.0044), confirming the robustness of the association (Supp. Fig. S5).

####  < 3 versus ≥ 3

In total, 19 studies [10–13, 18–21, 24–34], involving 4896 patients and 415 AL cases were included in the comparison between fewer than three SFs (1 + 2 SFs) and three or more firings. The analysis revealed a significantly lower risk of AL in patients with fewer than three firings. The fixed-effect model produced an OR of 0.40 (95% CI: 0.32–0.50, *p* < 0.0001), while the random-effects model estimated an OR of 0.38 (95% CI: 0.30–0.48, *p* < 0.0001). Between-study heterogeneity was negligible (I^2^ = 0%) (Fig. 2). No significant funnel plot asymmetry was observed (Egger’s test: *p* = 0.5137). The trim-and-fill method identified one potentially missing study, but the corrected OR remained consistent (OR: 0.39, 95% CI: 0.31–0.50, *p* < 0.0001), supporting the robustness of the findings (Supp. Fig. S6).

### Meta-regression results

**Fig. 3:**
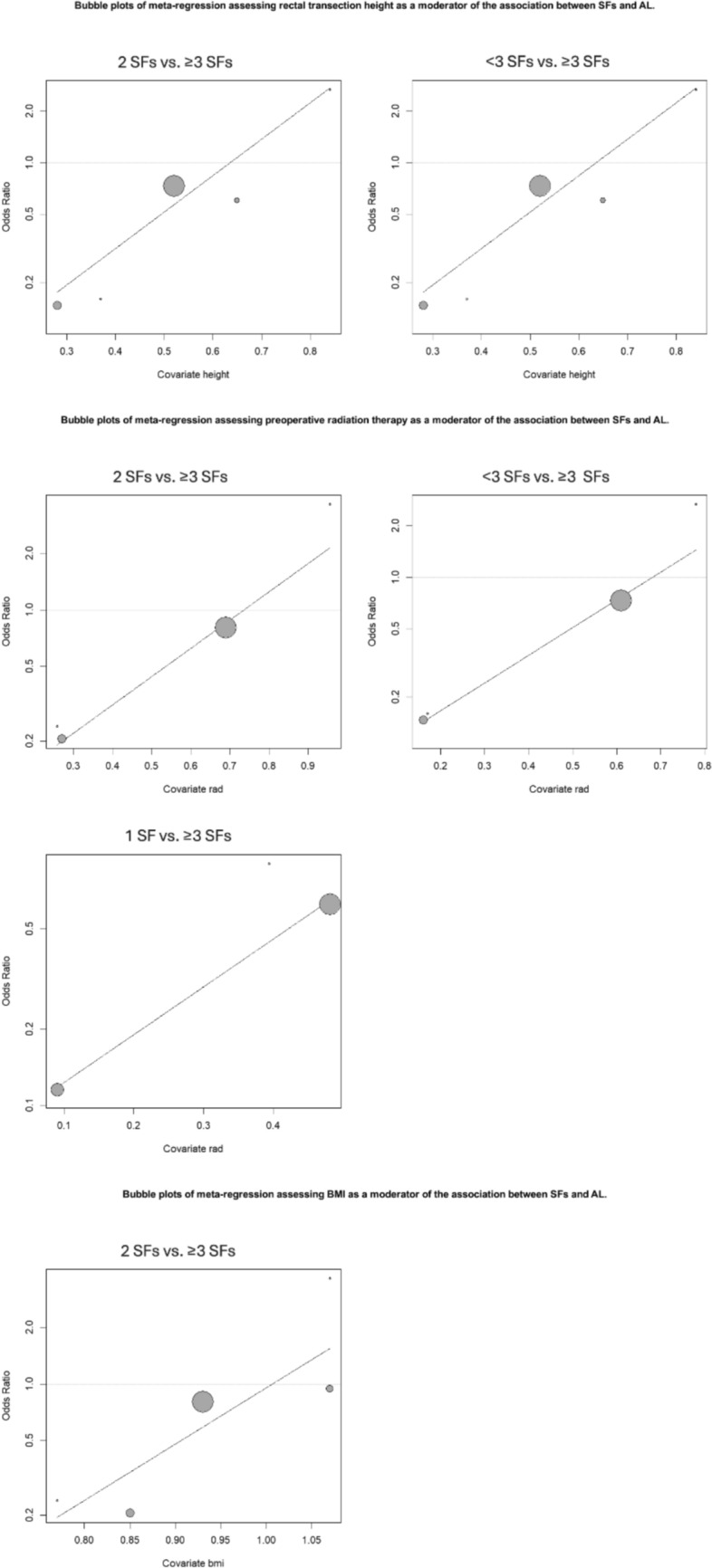
Bubble plots of meta-regression assessing moderators of the association between SFs and AL

Mixed-effects meta-regression was used to assess how study-level covariates—specifically patient rectal transection height, body mass index (BMI), and male sex, and preoperative radiotherapy—impact the relationship between the number of SFs and AL. For the comparisons of 2 versus ≥ 3 SF and the < 3 versus ≥ 3 SFs groups, when anastomotic height was included as a moderator, the model showed a significant positive association (β = 6.44, 95% CI: 0.52–12.37, *p* = 0.033). This suggests that patient subpopulations with lower rectal transection heights have higher odds of AL as the number of SFs increases (Fig. 3). The model reported an R^2^ of 100%, with a residual I^2^ of 0%, consistent with the low heterogeneity observed across studies. Preoperative radiotherapy emerged as a significant moderator across all three SF group comparisons. In the 2 versus ≥ 3 SF model, radiotherapy was significantly associated with increased odds of AL (β = 3.72, 95% CI: 1.13–6.31, *p* = 0.0049). Similar results were observed in the < 3 versus ≥ 3 SF comparison (β = 3.47, 95% CI: 0.53–6.40, *p* = 0.0207), and in the 1 versus ≥ 3 SFs comparison (β = 4.36, 95% CI: 0.68–8.04, *p* = 0.0203). The model reported an R^2^ of 100%, with a residual I^2^ of 0%, consistent with the low heterogeneity observed across studies (Fig. 3). However, meta-regression analyses were based on a small number of studies for each covariate (Fig. 3) and should therefore be considered exploratory. In this setting, the observed R^2^ = 100% and residual I^2^ = 0% likely reflect sparse data and very low between-study heterogeneity, rather than a stable estimate of explained heterogeneity.

Although the meta-regression models evaluating BMI as a moderator in the 2 versus ≥ 3 SF group explained a substantial portion of heterogeneity (R^2^ = 75.2%), the association between BMI and AL was not statistically significant (β = 6.91, 95% CI: −0.53 to 14.35, *p* = 0.069) (Fig. 3). Meta-regression for male sex did not produce statistically significant results.

### Sensitivity analysis

In the ISREC-only sensitivity analysis comparing 1 stapler firing versus ≥ 2 stapler firings, six studies (5159 patients) were included. A lower number of stapler firings remained significantly associated with a reduced risk of anastomotic leakage (random-effects OR 0.47, 95% CI 0.32–0.69), with moderate heterogeneity (I^2^ = 48.7%). Nevertheless, sensitivity analysis comparing ≤ 2 stapler firings versus ≥ 3 stapler firings (five ISREC-based studies with 1807 patients) again showed a significantly increased risk of anastomotic leakage with three or more stapler firings (random-effects OR 0.39, 95% CI 0.23–0.64), with moderate-to-low heterogeneity (I^2^ = 41.1%).

After exclusion of studies including mixed cohorts with open procedures, the association between increasing stapler firing number and anastomotic leakage remained consistent across all comparisons. In the random-effects model, the pooled ORs were 0.57 (1 versus 2 SFs), 0.36 (1 versus 3 SFs), 0.59 (2 versus 3 SFs), 0.54 (1 versus ≥ 2 SFs), and 0.42 (< 3 versus ≥ 3 SFs). Between-study heterogeneity was negligible (I^2^ = 0% in most comparisons), supporting the robustness of the primary findings in a purely minimally invasive population.

### Risk of bias

The GRADE assessment is presented in Supplementary Table S1, indicating low quality of evidence. Risk of bias was judged at the body-of-evidence level informed by ROBINS-I assessments of included studies and by the plausibility of residual confounding. Because key, study-level confounders (notably low rectal transection height and preoperative radiotherapy) plausibly affect both the exposure (greater number of stapler firings) and the outcome (anastomotic leakage), we applied a two-level downgrade (very serious) for the risk-of-bias domain across comparisons.

## Discussion

This comprehensive meta-analysis of 22 studies involving 8725 patients shows a strong link between an increasing number of SFs and a higher rate of AL after rectal resection. In all comparisons, especially < 3 versus ≥ 3 SFs, the data consistently reveal a statistically significant and clinically relevant rise in AL rates associated with an increased number of firings. However, our meta-regression results suggest that the height of rectal transection and previous chemoradiation therapy may affect this relationship. Notably, both lower rectal transection height and preoperative radiotherapy stood out as key moderators, which were individually linked to increased odds of AL in cases with multiple SFs. These findings raise the important question of whether such factors are not only effect modifiers but also potential confounders, especially since low rectal transection and transection of irradiated tissues are both technically more challenging and often require additional firings owing to limited working space, tissue fragility, and fibrosis.

The relationship between stapler firings and anastomotic leakage has been previously investigated. However, most available studies are single-center retrospective series, often limited to dichotomous comparisons (typically 1 versus ≥ 2 SFs), with heterogeneous definitions of anastomotic leakage and without a formal exploration of effect modification. The present study expands on previous literature by providing a comprehensive evaluation across multiple firing thresholds (1, 2, and ≥ 3), incorporating a substantially larger pooled population, and, importantly, applying meta-regression to explore the role of key contextual variables such as rectal transection height and preoperative radiotherapy. This approach allows us to move beyond a purely associative interpretation and to frame the number of stapler firings as a potential marker of operative complexity rather than a standalone causal risk factor, thereby offering previously unavailable insight into how this variable should be interpreted in clinical practice.

Our results build upon previous meta-analyses and offer a more detailed and nuanced understanding of the link between SFs and AL. Specifically, the earlier meta-analysis by Balciscueta et al. [6] focused on comparing one versus two firings in laparoscopic rectal surgery and found that two firings were associated with a significantly increased risk of AL (OR 2.44, 95% CI: 1.34–4.42, *p* = 0.003). Our findings confirm and reinforce this association while also extending the analysis to higher firing thresholds (e.g., ≥ 3 SFs), including a larger and more diverse patient cohort including both laparoscopic and robotic procedures.

Furthermore, unlike Balciscueta’s analysis, our study used meta-regression techniques, which revealed that the observed relationship could be significantly affected by contextual factors such as rectal transection height and preoperative radiotherapy—both of which were independently associated with higher AL risk when multiple SFs were employed. These findings emphasize the importance of interpreting stapler use not merely as a simple modifiable technical variable but as a combined indicator of operative complexity, especially in anatomically or oncological challenging cases.

There is no clear evidence regarding the etiopathogenesis of anastomotic discontinuity in AL [36]. No case series or literature exists where a root cause analysis has been performed with visual and tissue-level evidence to identify the underlying pathogenesis of AL. Recent preclinical and translational studies suggest a potential role of gut microbiota dysbiosis, dietary factors, nonsteroidal anti-inflammatory drug (NSAID) use, and pathogen-mediated collagenolysis in impaired anastomotic healing, although these findings are limited by the fact that they are not derived from human studies [37–39]. Current evidence mainly focuses on risk factors indirectly derived from retrospective analysis. Patient-related characteristics, such as male sex and BMI, surgical parameters, including anastomotic height, and other contributors, such as preoperative radiation therapy, have emerged as predisposing conditions [40, 41].

Moreover, these same factors provide both a technical reason for requiring more SFs and a potential risk for AL. Six studies [10, 11, 23–25, 33] analyzed the number of SFs in a multivariable system in relation to these other risk factors, showing variable results, with SF losing or maintaining significance depending on the study. Notably, De Giuli et al. [22] reported the number of SF as an independent risk factor for AL, along with anastomotic height, BMI, and male sex.

Therefore, while multiple SFs might indicate technical difficulty and increased tissue trauma, the higher risk of leakage could be due to the complexity of low pelvic surgery itself rather than the number of SFs. Conversely, if the increased interactions caused by more SFs are the main reason for AL development, then setting a limit range on the number of SFs should become a standard. Therefore, single stapling techniques, such as TTSS and glove technique [42–44], may represent a more attractive and potentially safer alternative.

Whether interpreted as a marker of operative complexity or a causative technical factor, the strong association between an increasing number of stapler firings and anastomotic leakage warrants heightened intraoperative vigilance. Although meta-regression indicates that this relationship is largely influenced by established risk factors, such as low anastomotic height and preoperative radiotherapy, a higher number of firings consistently identifies patients at increased risk of clinically relevant leakage. In this setting, diverting stoma should be primarily considered as a strategy to mitigate the severity and septic consequences of leakage rather than to prevent its occurrence, particularly when multiple firings are required despite a negative intraoperative leak test. Additional elements of a risk-adapted intraoperative bundle may include selective pelvic drainage, reinforcement of the anastomosis, and a lower threshold for intensified postoperative surveillance. Together, these measures support individualized decision-making in high-risk anastomoses.

While this study offers valuable insights, several limitations should be recognized. All included studies were observational, inherently carrying both confounding and selection bias, as indicated by ROBINS-I. Limited access to patient data restricted the scope of analysis and meta-regression, particularly regarding interaction effects. Data on stapler, cartridge length, and load type was inconsistently reported, mostly not stratified by AL or SFs, making its impact hard to assess—these findings should be viewed as descriptive. Use of diverting stoma varied and was influenced by patient and procedure factors, but inconsistent reporting prevented evaluating its effect on leakage. Intraoperative air leak testing was inconsistently reported, limiting assessment of its role. Surgeon-level technical factors were not reported and could not be analyzed directly. Limitations in dissection of the lower rectum are typically owing to anatomy and treatment constraints, such as narrow pelvis, obesity, radiotherapy, and low transection height, which were examined in meta-regression. Differences in definitions and grading of AL, as well as SF thresholds, across studies likely contributed to increased heterogeneity. Although both laparoscopic and robotic approaches were examined, outcomes could not be separated by approach owing to scarce subgroup data, which may influence stapler ergonomics and precision.

In conclusion, this meta-analysis shows a consistent association between an increasing number of stapler firings during rectal transection and higher rates of anastomotic leakage, particularly when three or more firings are required. However, because the certainty of evidence was low across all comparisons and due to the exploratory meta-regression analyses, SF number could be interpreted primarily as a marker of operative complexity rather than as a definitively proven independent causal risk factor. Efforts to minimize stapler firings remain technically reasonable, but the clinical relevance of firing number should be considered in the broader context of low rectal transection, preoperative radiotherapy, and other established risk factors for leakage.

## Supplementary Information

Below is the link to the electronic supplementary material.

Supplementary file1 (DOCX 291 KB)

## Data Availability

No datasets were generated or analyzed during the current study.
