# Supplementary material for: The impact of the number of stapler firings on anastomotic leakage in minimally invasive rectal surgery: risk factor or technical marker of complexity? A systematic review, meta‑analysis, and metaregression
Source: Tech Coloproctol. 2026 May 26;30(1):123. doi: 10.1007/s10151-026-03364-y (PMC13391776; doi:10.1007/s10151-026-03364-y)
Supplement: Supplementary file 1 — Supplementary file1 (DOCX 291 KB) [file 10151_2026_3364_MOESM1_ESM.docx]

Supplementary Fig. 1 Risk of bias domains


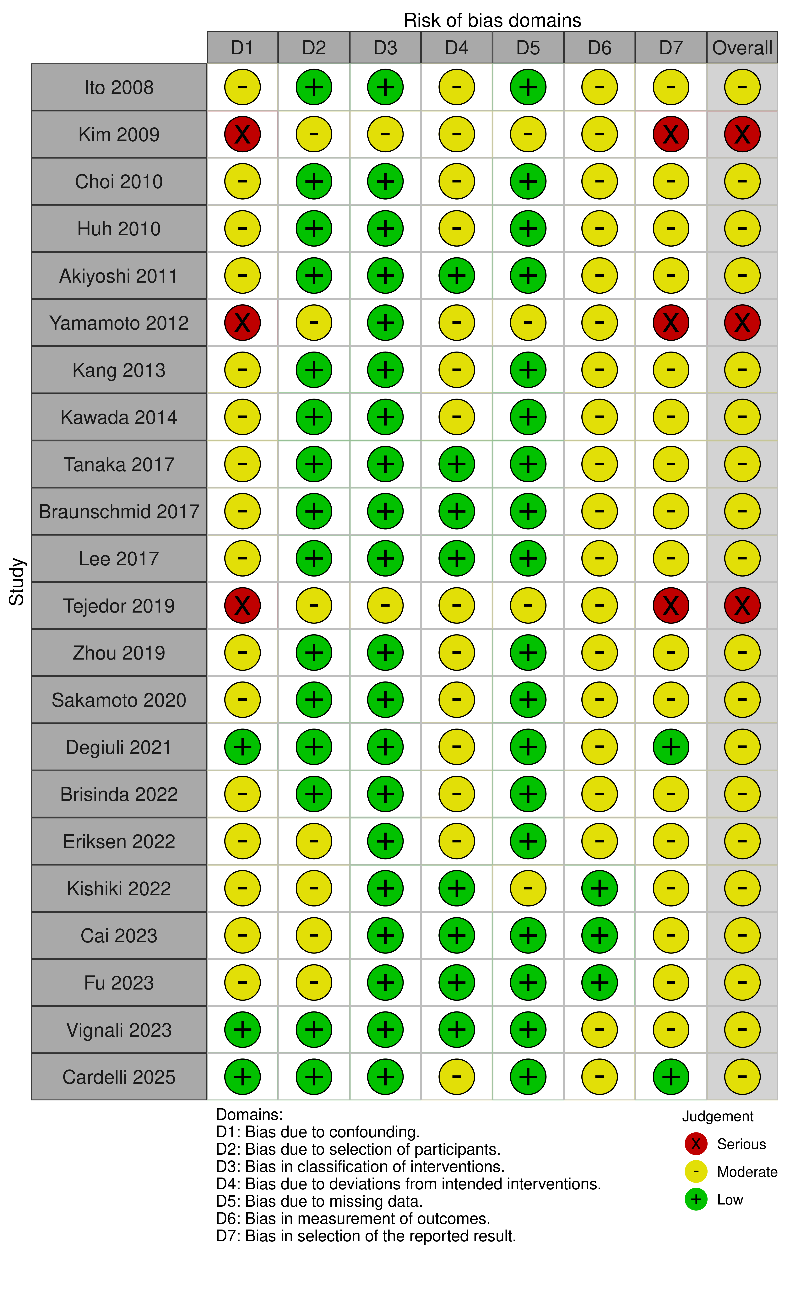


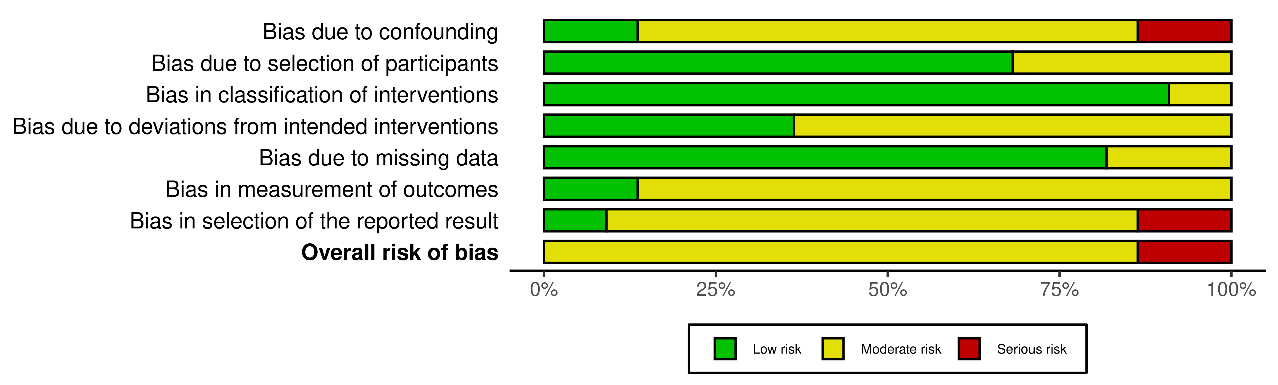


Supplementary Fig. 2. Funnel plot assessing publication bias for the comparison between 1 and 2 SFs.


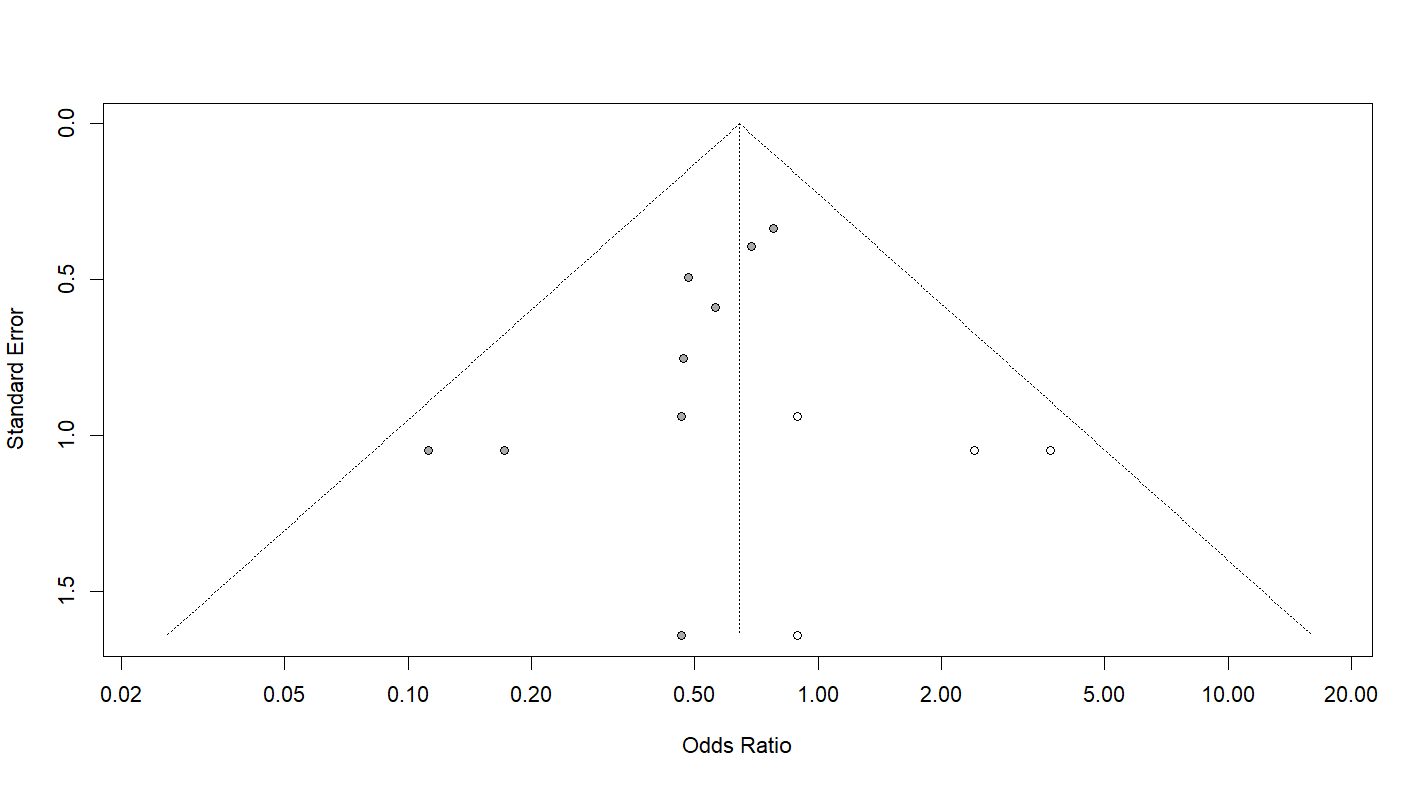


Supplementary Fig. 3. Funnel plot assessing publication bias for the comparison between 1 and 3 or more SFs.


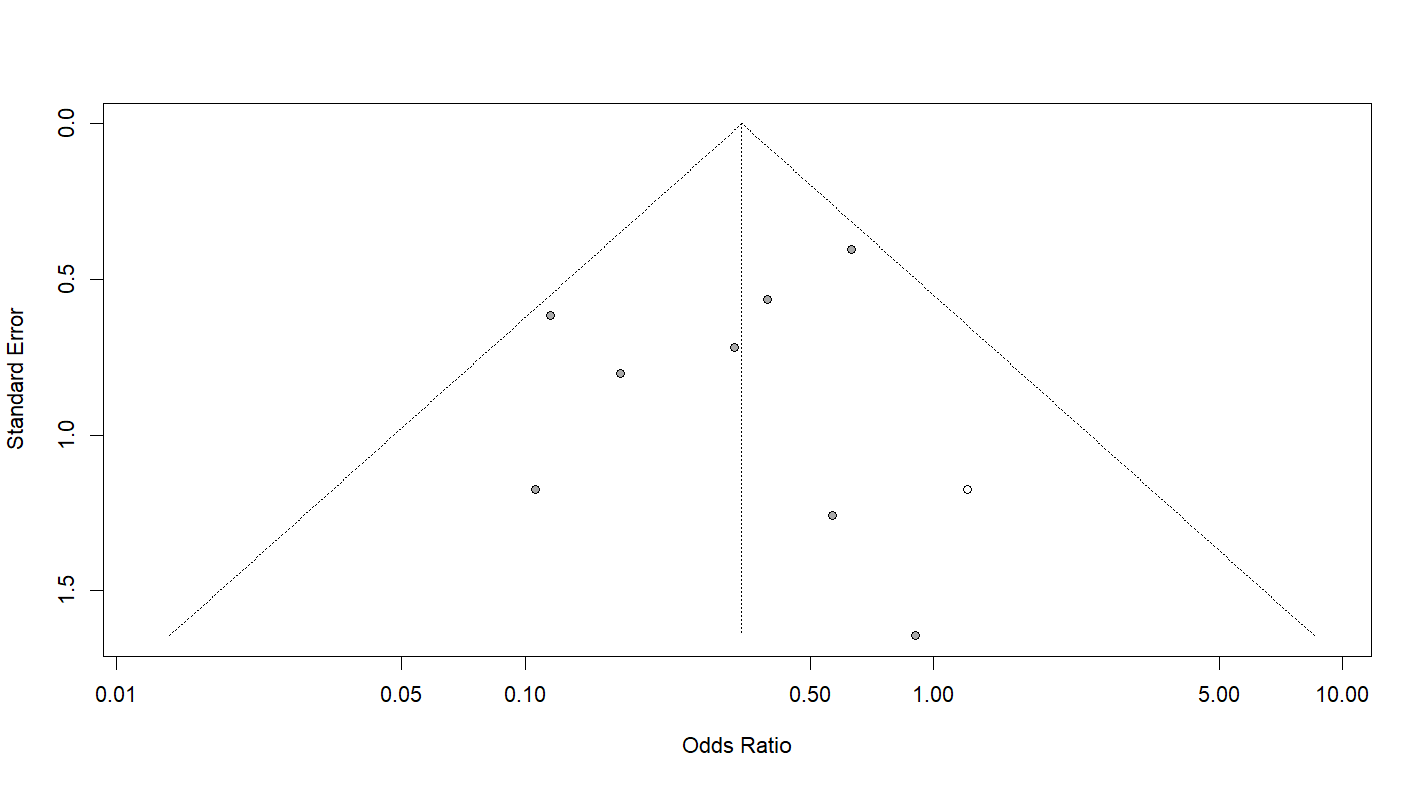


Supplementary Fig. 4. Funnel plot assessing publication bias for the comparison between 2 and 3 or more SFs.


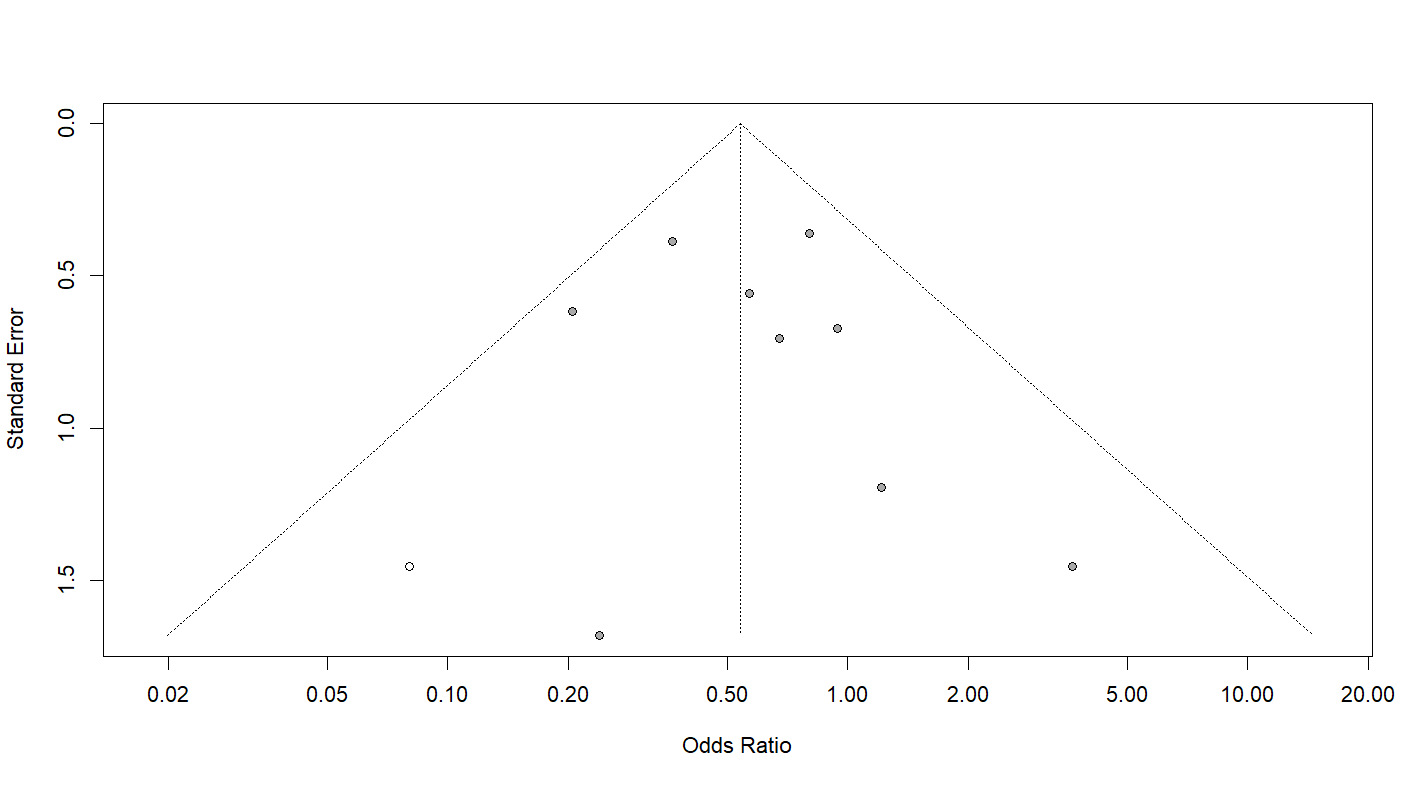


Supplementary Fig. 5 Funnel plot assessing publication bias for the comparison between 1 and more than 1 SFs.


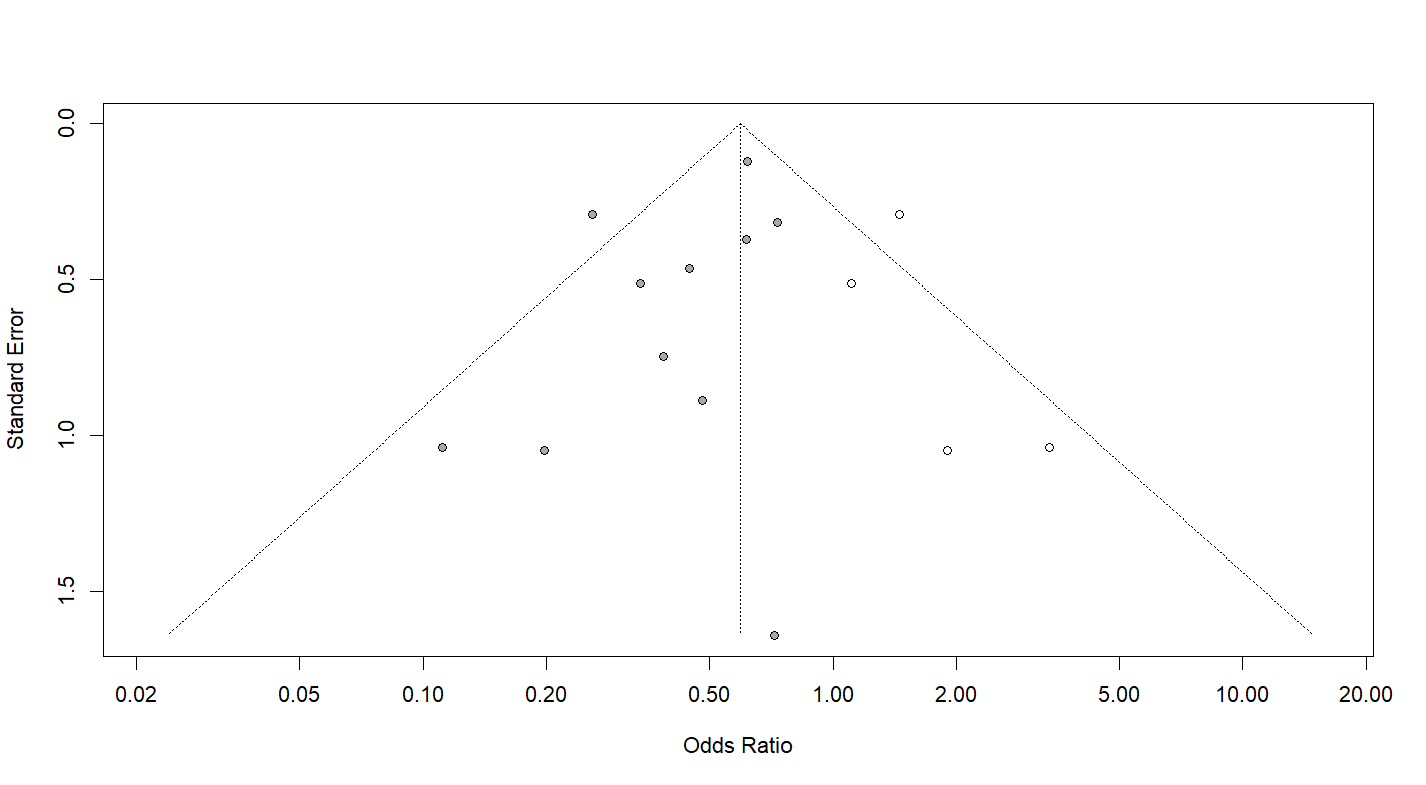


Supplementary Fig. 6 Funnel plot assessing publication bias for the comparison between less than 3 and more than 2 SFs.


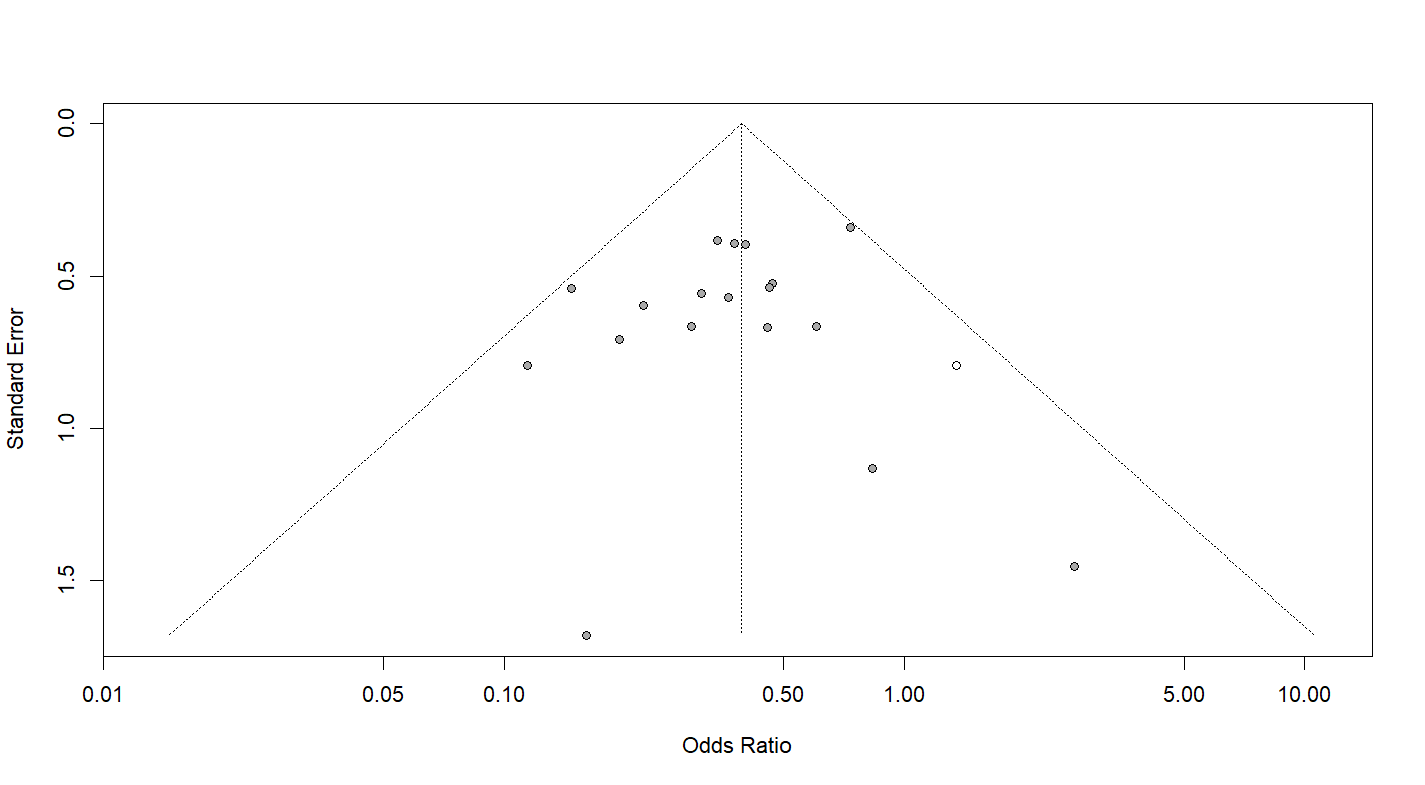


Supplementary Tab 1. Summary of Findings (GRADE) — Stapler Firings vs. Anastomotic Leakage

| Comparison | Outcome | Effect (OR, 95% CI) | Participants (studies) | Certainty (GRADE) | Reasons for rating |
| --- | --- | --- | --- | --- | --- |
| 1 vs. 2 stapler firings | Anastomotic leakage | 0.57 (0.39–0.83) | 2,781 (9) | Low | Risk of bias: very serious (−2) due to plausible residual confounding (lower transection level, pre-op radiotherapy) affecting both exposure and outcome; Inconsistency: not serious (I²≈0%); Imprecision: not serious (CI excludes 1, clinically narrow); Indirectness: not serious (population/intervention/comparator/outcome aligned); Publication bias: not serious (trim-and-fill did not change conclusions). |
| 1 vs. 3 stapler firings | Anastomotic leakage | 0.28 (0.16–0.51) | 2,781 (9) | Low | As above; Inconsistency: not serious (I²≈9%); trim-and-fill suggested at most one study; corrected effect remained significant. |
| 2 vs. 3 stapler firings | Anastomotic leakage | 0.53 (0.35–0.81) | 2,781 (9) | Low | As above; Inconsistency: not serious (I²≈0%); trim-and-fill suggested ≤1 study; corrected effect remained significant. |
| 1 vs. ≥2 stapler firings | Anastomotic leakage | 0.46 (0.34–0.63) | 6,610 (12) | Low | As above; Inconsistency: not serious (I²≈24%); Publication bias: not serious (Egger p≈0.10, trim-and-fill did not change the conclusion). |
| <3 vs. ≥3 stapler firings | Anastomotic leakage | 0.38 (0.30–0.48) | 4,896 (19) | Low | As above; Inconsistency: not serious (I²≈0%); trim-and-fill suggested ≤1 study; corrected effect remained significant. |

1. Approach: ROBINS-I–anchored GRADE for non-randomized studies; starting level High, then downgraded by domains.
